# Synthesis, Application and Effect of Hybrid Nanocomposites Based on Hydrogel and Nanoclay in Cement-Mortars

**DOI:** 10.3390/polym14214564

**Published:** 2022-10-27

**Authors:** Adhemar Watanuki Filho, Ricardo Tokio Higuti, Marcia Regina de Moura, Fauze Ahmad Aouada

**Affiliations:** 1 Department of Civil Construction, Federal Institute of Education, Science and Technology of Sao Paulo (IFSP), Alameda Tucurui, 164, Zona Norte, Ilha Solteira 15385-000, Brazil; 2 Department of Electrical Engineering, School of Engineering, Sao Paulo State University (UNESP), Avenida Brasil, 56, Centro, Ilha Solteira 15385-000, Brazil; 3 Grupo de Compósitos e Nanocompósitos Híbridos (GCNH), Department of Physics and Chemistry, School of Engineering, Sao Paulo State University (UNESP), Avenida Brasil, 56, Centro, Ilha Solteira 15385-000, Brazil

**Keywords:** civil construction, nanocomposite, polymer absorbent

## Abstract

Hybrid nanocomposite hydrogels, as admixtures for internal curing of cementitious materials, have been widely studied. This study analyzes the effect of applying 0.5% (wt/wt cement) of pre-soaked hydrogels based on polyacrylamide, carboxymethylcellulose, and three different concentrations of Cloisite-Na^+^ (0, 10, and 20% wt/wt) on the fresh and hardened properties of cementitious mortars. In general, all mortars with hydrogel decreased the consistency index, mainly M20, due to the high concentration of Cloisite-Na^+^ that modifies the release kinect of the hydrogel. The results showed a slight variation, with an overall average value of 99% water retention in all mortars. This behavior is due to the portion of hydrogel-mortars dosage water retained to reduce the availability of free water in the mixture because this amount of water is stored, a priori, within the polymer particles. At 28 d, the mortars produced with hydrogels containing 20% of nanoclay (M20) exhibit mechanical behavior similar to the reference mortar (M), which corroborates the percentage of voids found. Scanning electron microscope images confirm that the M and M20 mortars are uniform and possess few pores or microcracks. Thus, these hybrid hydrogels have the potential to be innovative materials for water control improvements in cementitious materials technology.

## 1. Introduction

Most cement-based materials consist of a binder matrix, with or without aggregates, and considered as porous composites [1] with good durability and versatility [2], which contribute to their widespread usage and study in the field of construction technology [3,4]. However, because these materials are heterogeneous and multiphase [5], they have properties that are directly affected by the characteristics of each constituent material [1]. Therefore, research on chemical admixtures has been carried out, mainly regarding water control [6], because most of the cement hardening chemical reactions occur in this component [7]. Water control is a method that is found to ensure the more effective hydration of cement particles, which contributes to the development of a more uniform microstructure and pore structure [8,9], improving the mechanical properties and durability [10] of cement-based materials. This procedure is denominated as an “internal cure”, and its function is not only to assist cement hydration but also to maintain high humidity in the cement matrix [11] to avoid small deformations, cracks, and low mechanical properties [12]. This procedure also enables the formation of a denser cementitious matrix because some cement-based materials may have porous microstructures that can facilitate the transport of water into the interior, causing chemical damage to the structure due to the transport of some ions [13]. It should also be emphasized that curing is an essential and recommended procedure for cementitious materials because, if correctly performed, it allows for potential gains in resistance and durability [14]. Examples of internal curing agents include materials such as lightweight-saturated aggregates [15] and absorbent polymers [6,16].

Thus, several studies on cement-based composites have been developed with the application of synthetic commercial superabsorbent polymers, based on acrylamide, acrylic acid, sodium acrylate, methyl acrylate, and so on, in cementitious materials as internal curing agents [17,18,19,20]. The main characteristics of these polymers are the absorption and release of water over time [21], which effectively contribute to the maintenance of the internal moisture of the cementitious matrix since traditional curing methods, such as wet and membrane curing, are considered external curing methods [9].

Recent research [9,22] has demonstrated the promising application of hydrogels in cement matrices, and understanding the behavior of polymers in cementitious materials is necessary for the optimum design of internal curing [23]. Hydrogels are also known as hydrophilic absorbent polymers. They are three-dimensionally cross-linked chain materials [24,25] synthesized from synthetic, semi-synthetic, or natural raw materials, whose main function is to absorb large amounts of water or other organic fluids [26] for later release. Some authors define hydrogels as polymeric systems that exhibit the capability to swell in water and retain a significant fraction (>20%) of water inside their three-dimensional structure, without dissolving in water [24,27]. The water storage of the hydrogels occurs from polymeric chain expansion due to repulsion from the hydrophilic groups on the polymeric chains, such as -OH-, -NH_2_-, -COOH-, -CONH_2_- and -SO_3_H- [28,29].

Importantly, the application of these hydrogels significantly changes the properties of cementitious materials because when applied presoaked, they change the rheology of mortars since part of the water is retained in the polymer matrix, and it introduces water into the curing process without the need to add a larger volume of water during the mortar mix [30]. However, this behavior is interesting because, with a more controlled release of water, the hydration of cement particles occurs more effectively, which may result in a reduction of voids in the microstructure and better mechanical properties.

This study is based on synthesis and the application of nanocomposite hybrid hydrogels based on acrylamide, carboxymethylcellulose (CMC) polysaccharide, and Cloisite Na^+^ nanoclay in cementitious mortars to allow the analysis of the effect of this polymer on the properties in the fresh and hardened states of these mortars. These polymers are composed of chemically, functionally, and morphologically distinct blocks, including natural, synthetic raw materials or nano/microstructures interconnected via physical or chemical means. They have been developed to improve existing formulations and to expand their range of applications [31].

The choice of these hybrid hydrogel types is based on the properties of each component for obtaining polymers with appropriate characteristics to be applied as internal curing agents for cementitious materials. Thus, this study attempted to develop a semi-synthetic hybrid nanocomposite based on acrylamide, a polysaccharide, and a mineral clay with hydrophilic and mechanical properties similar to commercial absorbent polymers. However, more controlled release kinetics [32] and the biodegradable feature can be applied as a potential internal curing agent in cementitious mortars.

The application of a polysaccharide, such as CMC, has been demonstrated to be a good option for the preparation of hydrogels because it has carboxylic groups in its chemical structure that can be ionized, and these groups have higher hydrophilicity than hydroxyl groups [33]. The choice for the use of clay minerals, such as Cloisite Na^+^, is justified by the fact that these are commonly applied at the nano- and microscale in the constitution of nanocomposites because they provide high thermal stability, good gas barrier properties [34], good mechanical resistance, and high degree of swelling and adsorption capacity [33]. However, the nature of how the nanoclay is dispersed in the hydrogel matrix can modify the behavior of the nanocomposite. Cloisite Na^+^ nanoclay in a polymer demonstrated that its releases kinetics becomes slower with increasing concentration in the polymer matrix [35]. The nanoclay presence in the polymeric matrix increases physical crosslinks, reduces the amount of water released overtime, and increases the release time of the hydrogels [36]. A nanoclay in the hydrogel composition can increase the process of water release [34] inside the cement matrix due to the improvements it provides for the hydrophilic properties of the hydrogel [37]. Therefore, using semi-synthetic hydrogels based on acrylamide, CMC, and Cloisite Na^+^ nanoclay may present similar or better behavior than commercial synthetics when applied in cementitious matrices, may make these polymers attractive to the construction industry as smart internal curing agents.

This study presents a comparative analysis of the influence of hydrogels with different nanoclay concentrations on the fresh and hardened properties of cementitious mortars. First, the swelling degree and kinetic parameters of the hybrid hydrogel nanocomposites are determined to aid in understanding the kinetics of water uptake and release from the polymer and how it may contribute to cementing hydration processes. The effects on the properties of the fresh state, such as consistency index, water retention and also in the hardened state, such as dynamic and elastic moduli, volume of permeable voids and Scanning electron microscopy (SEM and Energy-dispersive X-ray (EDX) spectroscopy analysis, are determined and discussed, with the aim of understand the interactions between inorganic and organic materials, which may help to control and adjust these properties. The guarantee that cementitious composites can have good physical and mechanical properties throughout their working life, including improvements in water retention [38], modifications in workability [39], and reductions in shrinkage and cracking by water evaporation [40] from the effective hydration of the cement particles, assumes that it is necessary to establish greater control over the availability of water in the cement matrix [6]. This condition can be remedied by applying a controlled release absorbent polymer.

It is expected that the addition of hydrogel nanocomposites will bring advances and improvements in the properties of mortars and that these materials can be used as additives in civil construction in the future.

## 2. Materials and Methods

### 2.1. Hydrogel Synthesis

Three types of hybrid nanocomposite hydrogels composed of polyacrylamide (PAAm), biodegradable polysaccharide carboxymethylcellulose (CMC), and Cloisite Na^+^ nanoclay were obtained through free radical polymerization, following the procedures described by Nascimento et al. [35] and Aouada et al. [41].

These hybrid nanocomposite hydrogels were synthesized using 6.0% (wt/v) acrylamide (AAm) monomer (Sigma-Aldrich, St. Louis, MO, USA, 99%, C_3_H_5_NO, MW = 71.08 g/mol) in an aqueous solution containing 1.0% (wt/v) of polysaccharide carboxymethylcellulose (CMC) (Synth, São Paulo, SP, Brazil, P.A, Mv = 114.000 g/mol), and different Cloisite Na^+^ (Southern Clay Products, Gonzales, TX, USA).contents: 0% (reference), 10% and 20% (mass% concerning to AAm + CMC mass).

It was used 2.0% (mol relative to AAm monomer) of *N*′-*N*′-metylenebisacrylamide (MBAAm) as crosslinking agent (Sigma-Aldrich, St. Louis, MO, USA, 99%, C_7_H_10_N_2_O_2_, MW = 154.17 g/mol), 6.67 mmol/L of *N*,*N*,*N*′,*N*′-tetramethylethylenediamine (TEMED) (Sigma-Aldrich, St. Louis, MO, USA, 99%, (CH_3_)_2_NCH_2_CH_2_N(CH_3_)_2_, MW = 116.20 g/mol) as reaction catalyst, and 3.50 mmol/L of sodium persulfate (Sigma-Aldrich > 98%, St. Louis, MO, USA, Na_2_S_2_O_8_, MW = 238.10 g/mol) as reaction initiator.

To improve the efficiency of hydrogel polymerization, nitrogen gas (N_2_) was necessary, after TEMED addition, for 10 min. After this stage, sodium persulfate solution was added under stirring into the polymeric solution to initiate the polymerization process. The hydrogel-forming solution was stored for 24 h at a temperature of 25 °C until complete polymerization.

Hybrid nanocomposite hydrogels were subjected to the dialysis process, i.e., changing the storage water daily for 7 days to eliminate the reagents not consumed. Subsequently, hydrogels were ground into microparticles and subjected to drying in an oven at 40 ± 1 °C for approximately 48 h or until achieving constant (variation < 0.50%). With the material completely dry, it was again ground and stored until its application. All concentrations of the required reagents were pre-established by our research group GCNH (Grupo de Compósitos e Nanocompósitos Híbridos) [42].

These hydrogels present values of swelling degree of 34.7 ± 1.9; 27.2 ± 1.3; and 24.4 ± 0.8 g·g^−1^ for 0%, 10% and 20% Cloisite Na^+^ nanoclay concentrations, respectively. The experimental procedure to determine these parameters will present in Section 2.3. The values are used to calculate the amount of water necessary to produce the mortars without modifying the water/binder ratio.

### 2.2. Mortar Preparation

Mortars-cement production followed the NBR 16541 [43] and NBR 7215 [44] recommendations. A composite cement type CPII-Z-32 (Ciplan Planalto Cimentos S. A.^®^, Brasilia, DF, Brazil) was used in their preparation because it is one of the most used types of cement in civil construction in Brazil. The physical and chemical compositions of the cement are presented in Table 1 and Table 2. The chemical composition was determined by X-ray fluorescence (XRF), according to Spósito et al. [45].

Siliceous sand (Castilho City, São Paulo-Brazil) was used as fine aggregate (fineness modulus of 2.05 and a specific gravity of 2650 kg·m^−3^) to produce all mortars.

The choice of the reference dosages proposed (Table 3) was based on the production parameters of the mortar for the test to determine the compressive strength of cylindrical samples proposed by NBR 7215 [44]. The variation of the type of hybrid nanocomposite hydrogel allowed to define 4 mortar systems, designated as M, M0, M10, and M20 where M represent the mortar control (without hydrogel or nanocomposite), and 0, 10, or 20 indicates the absence or the nanoclay amount into nanocomposites, respectively.

The hydrogel concentration (0.5% wt/wt in relation to cement mass) was determined according to the results obtained in previous researches [42]. It is important to mention that all the hydrogels were presoaked with water until the equilibrium state (48 h), to ensure that the hydrogel was completely charged with water when it was applied to the mortar. The amount of water absorbed by the polymer was removed from the dosage water to maintain the water cement ratio (w/c = 0.40) constant. It is noteworthy that no previous granulometry test was performed on the hydrogels after drying. This study is in progress. However, the particle size was adjusted to be similar to that of the sand used.

For the preparation of all mortars, a mechanical mixer (total volume 20 L) was used for 5 min. First, cement, water, and presoaked hydrogel were mixed for 60 s with minimum rotation (125 rpm around the main axis and 62 rpm of the planetary rotation). Without stopping the mortar mixer, all sand quantity was added for 30 s, and the rotation was increased to maximum (220 rpm around the main shaft and 125 rpm planetary rotation) for more than 30 s. After this agitation period, the mortar mixer was then stopped to remove and mix the materials adhered to the sidewalls of the container. The mortar remained at rest for 60 s, and after, it was remixed at maximum rotation for 90 s.

The mortars were cast in different molds according to tests realized. They were manually or mechanically densified (vibrated table SOLOTEST^®^, São Paulo, SP, Brazil) to eliminate incorporated air bubbles during the mechanical mix.

### 2.3. Swelling Degree (SD)

The hydrophilic properties of nanocomposite hydrogels were determined by measuring their swelling degree (SD). The determinations were measured at room temperature (25 ± 1 °C) by gravimetric analysis on an analytical balance (Shimadzu AUY-220-I). After the synthesis and dialysis procedures, the swollen hydrogels were cut into a cylindrical shape (circles of 22 mm diameter) and dried in an oven at 40 ± 1 °C until their constant mass. After, the dried hydrogels were placed into a vessel containing 20 mL of distilled water. The swelling measurements were performed in triplicate for each hydrogel-type analyzed.

For each predetermined time (measurements every 1 h up to 8 h, then at 24 h, 32 h, 48 h, and 72 h), the samples were withdrawn from swelling media, and the excess of the water surface was removed with soft paper. Then, their weights were measured using an analytical balance (Shimadzu AUY-220-I). Immediately, the samples were again placed on the vessel. The swelling degree was determined by the ratio between the mass of swollen hydrogel at the determined time and the dry hydrogel mass, according to Equation (1).

(1) SD=MtMd g H2O or solution per g hydrogel

where *M_t_* and *M_d_* are the weight of the swollen and the dried hydrogel, respectively. The measures were performed in triplicate (*n* = 3), and the error bars in the graph correspond to the standard deviation.

### 2.4. Kinect Parameters

Kinect parameters are obtained through swelling degree measures as the function of time (F vs. *t*) in different solutions. For each F vs. *t* curve, the diffusion exponent (*n*) and diffusion constant (*k*) were calculated using Equation (2) [47].

(2) MtMeq=ktn

where *M_eq_* is the hydrogel mass at equilibrium time, *t* is the time, *k* is a the diffusion constant (dependent on hydrogel type and swelling medium), and *n* is known as diffusion exponent that supplies the kind of water absorption mechanism.

Equation (2) was applied from the initial stage until 60% of the curve. Thus, the kinetic parameters involved in the mechanism of diffusion of water towards hydrogel were determined by the slope and linear coefficients of the ln (*M_t_*/*M_eq_*) *versus* ln(*t*) curve, respectively.

### 2.5. X-ray Diffraction (XRD)

X-ray diffraction (XRD) patterns of the clay, hydrogel, and their nanocomposites were obtained by (Shimadzu–XDR–6000) diffractometer using Cu-Kα radiation (λ = 0.154 nm) under a voltage of 30 kV and current of 40 mA. All specimens were analyzed in continuous scan mode with 2θ ranging from 5° to 50° at a scanning rate of 1° min^−1^. Additionally, the basal spacing or the distance of two adjacent nanoclay platelets was determined from the position of d (001) reflection, which is calculated by Bragg’s equation (n λ = 2dsinθ).

### 2.6. Mortar Consistency Index

The determination of the consistency index was based on the ASTM C1437 [48].This technique permitted to analyze the influence of the nanocomposite hydrogels on the fresh properties of the hybrid mortars. The testing was performed immediately after the hybrid-mortars mixing. The addition of the fresh mortar into the trunk-conical mold on a flow table (CONTECO^®^, São José da Lapa, MG, Brazil) occurs in three layers. The first layer was densified with 15 strokes randomly distributed in the mass. The second and third layers were densified with 10 and 5 strokes, respectively. The excess mortar was removed, and the mold was vertically removed. Finally, 30 strokes were applied to the flow table for 30 s. Thus, three measures of the diameter of the mortar on the table were obtained. The consistency index average was calculated by the three diameters obtained from its scattering.

### 2.7. Water Retention

Water retention capacity in fresh mortars is very important because the available water inside the mix increases. It can improve the cement hydration and, consequently, to obtain a mechanical strength gain. Thus, water retention analysis in hybrid mortars is realized because the hydrogels can act as water retentor agents. This study followed EN 1015-8 [49] recommendations, testing six samples of each fresh mortar.

After mortar preparation (35 ± 2 °C and relative humidity ~55%), a fraction was collected and cast inside the cylindrical mold (*m*_1_) with a diameter of 100 mm and a height of 25 mm. The excess mortar was then removed from the top of the mold, and the mortar + mold set mass (*m*_3_) was recorded using a semi-analytical balance (Shimadzu BL-3200H).

Subsequently, gauze and filter papers (*m*_2*i*_)—weighed previously in a dry state—are placed on the set so that on a flat surface and in an inverted position, a load of 2 kg is placed on the stage for 5 min. The filter paper was removed and again weighed (*m*_4*i*_). Thus, the water retention is determined following Equation (3).

(3) $$WRV_{i}=100-W_{4i}$$

where *WRV_i_* = water retention of mortar sample “*i*” (%); *W*_4*i*_ = relative water loss by mortar “*i*” (%), calculated by using Equation (4).

(4) W4i=m4i −m2im5i −W1i∗100%

where *m*_5*i*_ = amount of paste “*i*” inside the mold (g), calculated by using Equation (5), and *W*_1*i*_ = total water contained in paste “*i*” (g), calculated by using Equation (6).

(5) $$m_{5i}=m_{3i}-m_{1i}$$

where *m*_3*i*_ = mold mass containing paste “*i*” (g); *m*_1*i*_ = mass of the mold associated with paste “*i*” (g).

(6) W1i=mwater “i”mmortar “i”

where *m_water_*_“*i*”_ = total water mass used to produce the mortar “*i*” (g); *m_mortar_*_“*i*”_ = total mortar mass “*i*” (g); com *i* = 1, 2, 3, …, *n*.

### 2.8. Dynamic Elastic Modulus

Dynamic elastic modulus was found by using prismatic molds (40 × 100 × 10 mm). The samples were removed from the mold after 24 h. Six samples of each characteristic age were cast and stored under the same curing conditions (ambient curing in a room with relative humidity ~55% and temperature of 35 ± 2 °C), as described in the previous procedure, until 48 h before the test. Immediately before the measures, these samples were dried in an oven (Quimis Q317M52, São Paulo, SP, Brazil) at 40 ± 1 °C for 48 h.

Dynamic elastic modulus was obtained using a 100 V pulsator/receiver assembly system, 500 kHz transducer frequency, and digital storage oscilloscope (TDS 2022). Finally, the measures were realized in two different regions of the prismatic sample to obtain the mortar behavior throughout the specimen. All procedure was based on the ASTM C597-16 [50], and it was determined at 7, and 28 d ages by applying Equation (7).

*Ed* = *v*^2^ × *ρ* × ((1 + *μ*) × (1 − 2*μ*)/(1 − *μ*)) (7)

where *Ed* is the dynamic elastic modulus (GPa), *v* is the ultrasonic velocity (m/s), *ρ* is the bulk density of mortars (kg/m^3^) and *μ* = coefficient of poison (*μ* = 0.20).

### 2.9. Elastic Modulus

The elastic modulus was determined for cylindrical molds (100 × 200 mm) with curing ages of 7, and 28 d. Twelve samples, in each age, were cast and maintained in curing conditions (relative humidity ~95% and 35 ± 2 °C) until the test age. This destructive test was carried out on an EMIC Universal machine (Instron Brasil Equipamentos Científicos Ltda, São José dos Pinhais, PR, Brazil) with a 200-ton load limit and loading rate of 0.25 ± 0.05 MPa/s, following the ASTM C469M-14 standard [51]. The strain gauges were used directly on the samples to determine the strain data.

### 2.10. Volume of Permeable Voids Spaces

Volumes of permeable void spaces were tested following ASTM C642-13 [52] at 28 days age. A total of six samples (Ø = 3 cm and height = 1.5 cm) for each mortar type were prepared, with three samples remaining under wet curing (relative humidity ~95% and 35 ± 2 °C), and the other three samples under ambient curing (35 ± 2 °C and 55% Relative Humidity) conditions until 28 days.

At 28d, the masses of the samples were determined on a semi-analytical balance (Shimadzu BL-3200H) and dried in an oven (Quimis Q317M52, São Paulo, SP, Brazil) at 105 ± 5 °C for 72 h. During this period, all masses were measured every 24 h.

After 72 h, the specimens were immersed in water (25 ± 2 °C) for another 72 h to determine the saturated mass after immersion, being again weighted every 24 h. Subsequently, the previously immersed specimens were placed in a suitable receptacle, covered with water, and boiled for 5 h. The samples were cooled by a natural loss of heat for 24 h, then their saturated masses after boiling were determined (Shimadzu BL-3200H).

Finally, after immersion and boiling processes, all specimens had their apparent masses determined on a hydrostatic balance. Then, an estimate of the volume of permeable voids was determined by Equation (8).

(8) %Vol.permeable pore space voids=(g2−g1)g2∗100

where *g*_1_ = bulk density, dry, Mg/m^3^ and *g*_2_ = apparent density, Mg/m^3^. * (1 Mg/m^3^ = 1 g/cm^3^).

### 2.11. Scanning Electron Microscopy (SEM) and Energy-Dispersive X-ray (EDX) Spectroscopy

The microscopic analysis was conducted using the R, N0, N10, and N20 mortars at 7 and 28d age. The samples were a small piece of cementitious mortar removed from the central region of the mortar samples. They were dried for 48 h in an oven (40 ± 2 °C). After, the surface was coated with a thin gold layer to avoid charging during SEM imaging. The micrographs of the analyzed samples were obtained using the ZEISS scanning electron microscope, model EVO/LS15, with an acceleration voltage of 20 kV.

The EDX technique identified the chemical elements in the mortars produced without and with nanocomposite hydrogels. An Oxford Instruments X-ray dispersive energy spectroscope, Inca X-act model with 100 eV resolution, coupled to the cited microscope was used.

### 2.12. Statistical Analysis

The experimental results for each treatment set were available by analysis of variance (ANOVA) from the Tukey test, with a 5% significance level, using SISVAR^®^ software, version 5.6.

## 3. Results and Discussion

### 3.1. Swelling Degree (SD)

**Figure 1:**
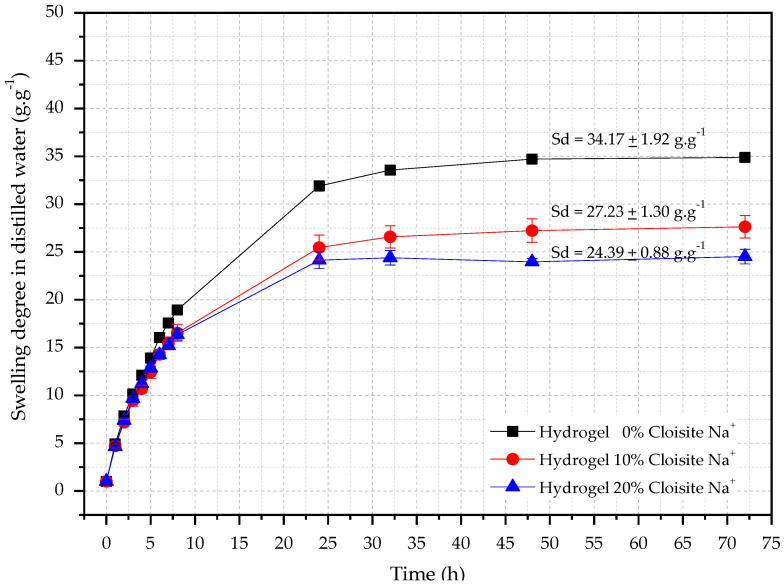
Swelling curves as a function of time for hydrogels with different Cloisite-Na^+^ concentration in distilled water.

Swelling degree (SD) analysis of hybrid hydrogels is necessary once the controlled water release over time depends on this property and can contribute to a more effective internal curing procedure of cementitious materials [53] due to the continuous cement [42] hydration inside of these materials. The absorption behaviors of hybrid nanocomposite hydrogels in distilled water are shown in Figure 1.

It is seen from Figure 1 that all hydrogels showed water absorption in the range of 24–32 (g·g^−1^) in distilled water. Also, the Cloisite-Na^+^ interferes directly in this hydrophilic property because the higher its concentration, the lower hydrogels swelling degree, corroborating with reported by Aalaie et al. [54]. The authors verified that the equilibrium degree of swelling of the nanocomposites decreases with the increase of montmorillonite content because the free hydrophilic groups of the nanocomposite reduce with the Na^+^ increases, decreasing the difference in the osmotic pressure between the matrix and the swelling medium and, consequently a retraction of the hydrogels with Cloisite-Na^+^ [54].

In the first eight hours of testing, it was possible to observe an accelerated water absorption, independently of the concentration of nanoclay, reaching the equilibrium conditions after 48 h. Similar behavior was also observed by Yenozawa et al. [55]. The results showed that the PAAm + CMC hydrogel had SD_equilibrium_ equal to 34.71 ± 1.92 g·g^−1^. For hybrid hydrogels with 10% and 20% Cloisite-Na^+^ concentration, the average SD_equilibrium_ were 27.23 ± 1.30 g·g^−1^ and 24.39 ± 0.88 g·g^−1^ respectively.

**Figure 2:**
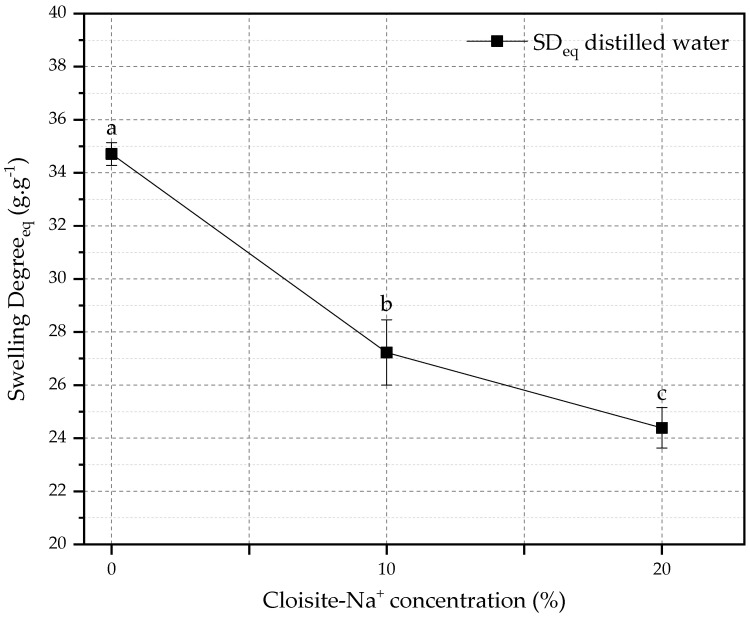
Effect of the amount of Cloisite-Na^+^ on the equilibrium swelling degree. Average with their respective standard deviation values, followed by equal letters do not differ statistically from each other following the Tukey test with a 95% confidence level.

The relationship between SD_eq_ and the amount of Cloisite-Na^+^ in the nanocomposites is shown in Figure 2. The reduction of SD_eq_ for hybrid hydrogels (10 and 20% Cloisite-Na^+^) was 21.55% and 29.73%, respectively, when compared with PAAm + CMC hydrogel. This behavior can be attributed to the presence of nanoclay as a physical crosslinker in the polymeric matrix, which can cause a reduction in the expansion capacity of the chain and in the water storage capacity in its pores [56,57].

These behaviors also can be attributed to their molecular structure and morphology [23] because the open spaces (pores) among the polymer network decreased, and the number of pores increased when the nanoclay concentration used in the hydrogel preparation increased, corroborating with Cojocariu et al. [56].

Thus, hydrogels with large pores interact more significantly with water molecules, resulting in large water uptake. On the other hand, Cloisite-Na^+^-based nanocomposites have a tighter structure with smaller pore sizes, and the area of the pores in contact with water molecules is small, which contributes to decreasing water-uptake capacity [58].

### 3.2. Kinect Parameters

The mechanism of water absorption is associated with the diffusional exponent (*n*) and the diffusion speed of the solvent (*k*) [58]. The mechanism can occur in four different ways: where *n* < 0.45, the mechanism occurs by Fickian diffusion; when *n* = 0.89, the diffusion occurs by case II-transport; in other words, this mechanism is governed by polymer swelling (chains relaxation); for 0.45 < *n* < 0.89, the diffusion mechanism is classified as anomalous transport (non-Fickian diffusion), that is, the combination of the two previous; and when *n* > 0.89 corresponds to super case II-transport [47,59,60,61]. Changes in kinetic parameters as a function of Cloisite-Na^+^ concentration are shown in Table 4.

All nanocomposites presented values of *n* above 0.45 and below the control matrix. The water absorption mechanism of all hydrogels has anomalous behavior, that is, when the diffusion times and relaxation rates of the chains are comparable. Thus, both sorption and transport of molecules are affected by the presence of pre-existing microcavities in the polymeric matrix [60]. However, the increase in the Cloisite-Na^+^ concentration in the nanocomposite matrices modifies the water absorption, tending to *Fickian* transport, where the diffusion rate is much slower than the relaxation time of the polymer chain. This relaxation time is the time it takes for the chain to settle, that is, to come into balance with the presence of the solute or solvent.

The presence of nanoclay in the polymer matrix increased the values of the constant diffusion *k*. The authors believe that the oxygen bound to the silicon of the nanoclay platelets may be simultaneously interacting with the groups of the CMC chain and with distilled water, accelerating the water absorption process [55]. However, it is intended that even accelerating the velocity of water absorption, the nanoclay also acts as a crosslinking agent, contributing to the decrease in total water absorbed by the matrix. These results corroborated with SD results, whereas these nanocomposites were those with less water absorption capability.

### 3.3. X-ray Diffraction (XRD)

**Figure 3:**
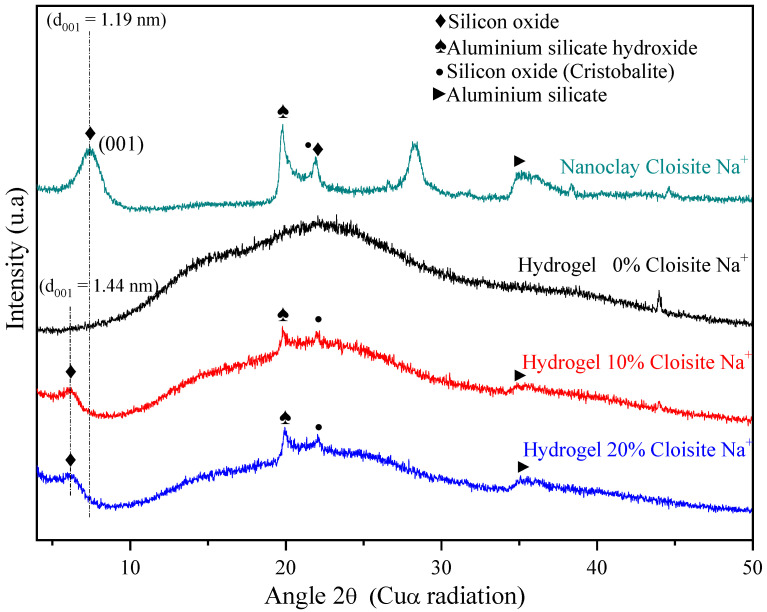
X-ray diffraction for PAAm/MBAAm/CMC + Cloisite Na^+^ swollen in distilled water.

XRD analysis of the Cloisite Na^+^ nanoclay (Figure 3) displayed a reflection at 2θ = 7.36° assigned to the (001) crystalline plane or d_001_ interlayer basal spacing of 1.19 nm, agreeing with other authors [62,63]. Besides, the reflections at 2θ = 19.8°, 21.9°, and 28.3° could also be observed, and they correspond to aluminum silicate hydroxide, silicon oxide, and aluminum silicate hydroxide [30], respectively.

XRD patterns of hydrogel pure (PAAm and CMC) and hybrid nanocomposite hydrogels (PAAm/CMC and Cloisite Na^+^) confirmed that these matrices are predominantly amorphous, as expected due to their chains have high crosslinking density. However, it is possible to observe, from hybrid hydrogels XRD, that the characteristic nanoclay peak (2θ = 7.36°) shifted to the small angle of the 2θ = 6.28°, causing an increase in basal spacing (d_001_ = 1.44 nm). This increase indicated the intercalation of nanoclay Cloisite Na^+^ in the polymer matrix, as mentioned by Romanzini et al. [64].

Wang and Wang [65] reported the main reason for nanoclay intercalation. They confirmed that CMC molecules were successfully intercalated into the spacing of clay layers, reducing their crystal structure. He et al. [66] also related that the interaction between AAm monomer and montmorillonite, during the polymerization process, can form a gel mesh structure that increases basal spacing. It is reported that the arrangement of clay layers results in a broad XRD pattern, broadening of the d_001_ peak observed in modified montmorillonite reflected a lower degree of ordering for montmorillonite layers than natural clay. Therefore, the interlayer structure of montmorillonite is damaged, and the weaking of the hydrogen bonding reduces its crystallinity [66].

### 3.4. Mortar Consistency Index

**Figure 4:**
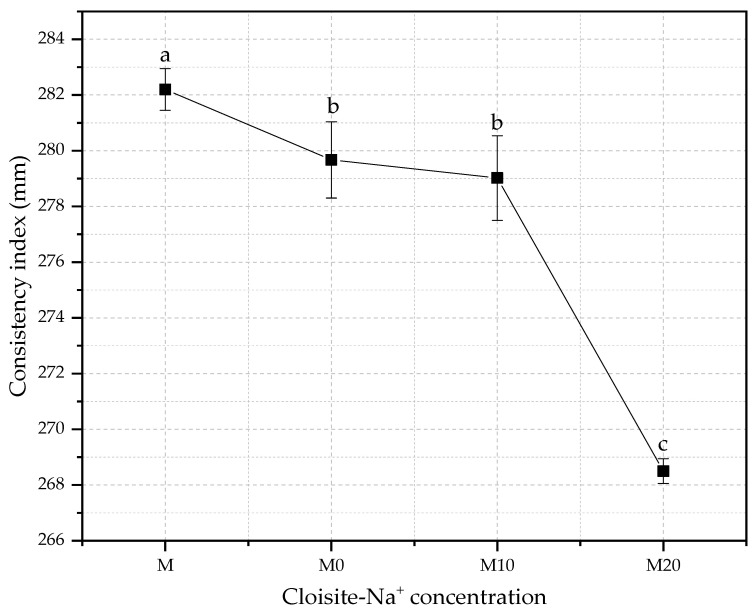
Comparative of the consistency index for mortars with different water/cement ratios different Cloisite-Na^+^ nanoclay hydrogels. Average with their respective standard deviation values, followed by equal letters do not differ statistically from each other, following the Tukey test with a 95% confidence level.

The consistency index is a parameter defined in function of the purpose of application of the cementitious material. Thus, this index measures the workability, which is considered one of the most important properties of cement-based materials, and a key, site-specific factor considered in mortar design [67]. The changes in consistency index for mortars produced with different hydrogel and nanoclay concentrations with w/c of 0.40 are shown in Figure 4.

The decreases in the slump flow regarding the control were 0.9%, 1.1%, and 4.9% for M0, M10, and M20 mortars. The reduction in free water in the fresh mixture will inevitably affect the workability of material cementitious [68], whereas an effective w/c ratio is lower than those proposed by the initial dosage because part of the free water remained in the absorbent polymer matrix.

Gupta et al. [69] also observed that the flow is significantly reduced even at higher superabsorbent SAP (sodium polyacrylate-based commercial) dosage as the amount of pre-absorbed water in SAP increases. Two main reasons are probably related: (i) the reduction in free water content in the fresh mix due to absorption of water by SAP; (ii) the swollen SAP particles, which act as soft aggregates. The results presented by these authors showed that 0.3% SAP-mortars reduced around 17% the values of slump when compared with reference mortar produced with cement, sand, and water (1: 2.75 w/c ratio = 0.45).

Similarly, Dang et al. [5] demonstrated that synthetic SAP based on sodium polyacrylate (Hebei Xiguang Chemical Technology Co., Ltd., Hengshui City, Hebei Province, P.R. China), presoaked with deduction of mixed internal curing water, also had a significant influence on the slump of concretes. They verified that concretes produced with 0.3% of SAP in their composition present a reduction of about 31% in their consistency index compared with reference samples, following the same trend as the obtained results. Yang et al. [70] also observed that cement-mortars with 0.4% of synthetic SAP (polyacrylic acid), concerning the cement mass, reduced its slump flow, attributing it to the thickening effect caused to SAP in the mixture due to the free water absorption by these polymers.

Senff et al. [71] showed that the presence of dry synthetic SAP (Evonik^®^, Essen, Germany) reduces the mortar workability, and this decrease is attributed to ionic nature and the interconnect chain structure of SAP particles that improve the chemical affinity with the water kneading molecules, increasing the level of absorption of these polymers. Consequently, the quantity of free water in the mix decreased, and it was necessary to adjust the w/c ratio to maintain the workability for a more extended period.

Another relevant aspect to be discussed about the results obtained is that besides hybrid nanocomposites having evident potential as hidroretentor agent, as also described by Paiva et al. [72], the Cloisite-Na^+^ nanoclay presence directly influences the absorption and release kinect of the hydrogel over time, impacting on the fluidity of cement-material in their fresh state.

This occurs because the nanoclay, besides acting as a reinforcing agent, it can improve some physical properties of polymers [56,73,74] and increase the hydrophilic properties of these nanocomposites [75]. Thus, the effect obtained by adding the nanoclay reduces the swelling degree since the clay intercalated into the polymeric chains can act as a crosslinking agent for these polymer networks and, consequently, a slower water release over time [35], reducing the workability when applied in cement-materials.

### 3.5. Water Retention

**Figure 5:**
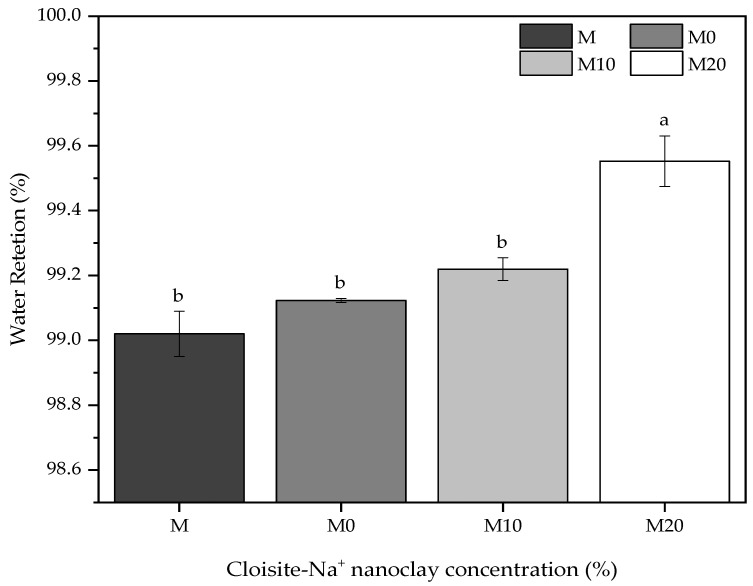
Comparative of the water retention for mortars containing nanocomposites prepared from different Cloisite-Na^+^ nanoclay amount and water/cement of 0.40. Average with their respective standard deviation values followed by equal letters do not differ statistically from each other, following the Tukey test with a 95% confidence level.

Water retention is a property associated with the capacity of fresh mortar to maintain its workability when subject to solicitations that occasioned kneading water losses either by evaporation or water absorption by the settlement substrate [76]. The importance of this property is mainly in its application aspects, influencing the adherence and productivity of those who apply it. Figure 5 shows the water retention for reference mortars and nanocomposite hybrid mortars, varying the Cloisite-Na^+^ nanoclay concentration in its polymeric matrix.

In general, the results presented a slight variation (but statistically significant) among them, with an overall average value of 99% in water retention for all mortars. Satisfactorily, these results are according to ASTM C270-19a [77], where the minimum retention is 75%, and the recommendations of NBR 13278 [78] that permit to classify of these mortars as “Class E” because they have water retention values between 95 and 100%.

As mentioned, applying hydrogels with Cloisite-Na^+^ nanoclay in these mortars increased this index, being more evident for M20 mortar, i.e., the highest nanoclay concentration in its polymeric structure. The hydrogels without and with 10% nanoclay in their compositions did not cause significant statistical effects for mortars compared with the reference sample M. These slight variations were 0.1% and 0.2% for M0 and M10 mortars produced with 0.5% (wt/wt cement) hydrogel, respectively. However, M20 mortar had an increase of 0.5% concerning M mortar, revealing that the nanoclay acts as an important agent in water retention by the polymer. This occurs because the polymeric structure of hydrogel modifies its morphology, acting, as already discussed, as a physical crosslinker and changing the absorption and release of water kinetics [29,79].

This behavior can be related to the water parcel of dosage of hydrogel-mortars presoaked to reduce the availability of free water in the mix. In contrast, this amount of water is stored, a priori, within the polymer particles [20]. Thus, an internal water reservoir is created into the fresh cement material, acting as a curing agent by gradually releasing absorbed water during the hydration process [80]. This trend was also observed by Tenório Filho et al. [38], who affirm that synthetic SAP hydrogels absorb and retain a certain amount of the water (depending on their absorption capacity) after the water reservoir acting, keeping its level of internal relative humidity high for a considerable time. Indeed, Jensen et al. [81] noted that water retention in SAP also reduces free water content and w/b ratio due to its capacity of absorption or retention part of the mixed water upon dosage in concrete.

Water retention of these mortars due to the hydrogel dosage directly affects the workability, corroborating with the consistency index results presented in Section 3.3. Although this direct reduction and modification in the rheological properties of the cement-materials related by several authors [71,82], water retention can be interesting because allows it allows internal humidity is maintained [83]. This gain may prevent cracks due to plastic shrinkage [84] and improve the hydration of cement particles.

### 3.6. Dynamic Elastic Modulus

**Figure 6:**
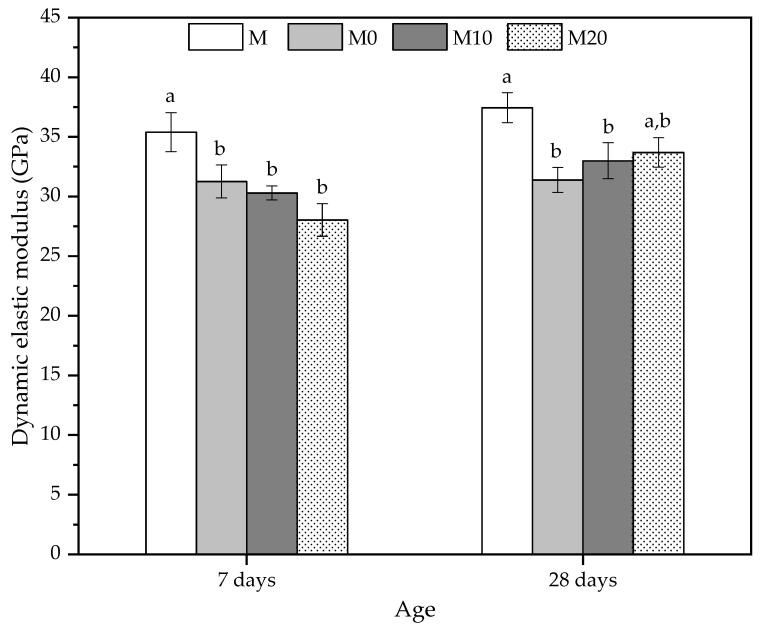
Dynamic elastic modulus at 7 and 28 days obtained by ultrasonic velocity to mortars produced with 0, 10, and 20% Cloisite Na^+^ nanoclay hydrogels. Average with their respective standard deviation values, followed by equal letters do not differ statistically from each other following the Tukey test with a 95% confidence level.

The mechanical behavior of the cement mortars was also investigated using ultrasonic non-destructive testing [85]. The dynamic elastic modulus results in Figure 6 indicated that all the mortars prepared with hydrogels have a lower average value at 7 d compared to the reference mortar (M).

At 7 d, the reductions were 11.69%, 14.41%, and 20.83% for M0, M10, and M20, respectively. Despite presenting a reducing trend for the dynamic elastic modulus, with the increase of nanoclay in the hydrogels, the mortars with hydrogels did not show statistically significant differences between them. The pre-soaked hydrogels reduce ultrasonic velocity, which may also be related to the decrease in the compressive strength values for this age [85]. The dynamic modulus results agree with the compressive strength results, whereas the mechanical strengths reduced this property and showed similar behavior.

At 28 d, the mortars with hydrogels also showed lower average values than the control. The reductions were 16.16% and 11.89% for M0 and M10, respectively, and they are smaller as the nanoclay concentration of the hydrogel increases. However, the result of the M20 mortar is statistically similar to the reference (M) mortar. As indicated in Figure 6, the average values of this matrix were 33.67 ± 1.24 GPa, showing that the nanocomposite increases its stiffness due to a possible improvement in matrix densification caused by a slower release process. This behavior is linked to the kinetics of water release by the polymer with a high amount of nanoclay, indicating that despite the lower swelling degree, this type of hydrogel [56] can release water for longer periods in the internal curing process. In addition, the Cloisite Na^+^ in the hydrogel may also act as a reinforcing agent for the microstructure of the mortar, mitigating the effects of porosity on the compressive strength and elastic modulus.

The discrete variation of the modulus represents a satisfactory condition because the polymer influences the chemical reactions initiated by the hydration of the Portland cement components [42]. Thus, these products obtained due to hydration for a longer period can compensate for the porosity formed by the hydrogel after its complete water-release process, thereby making the matrix more compact with less shrinkage and consequently without microcracking, as seen in the SEM images.

### 3.7. Elastic Modulus

**Figure 7:**
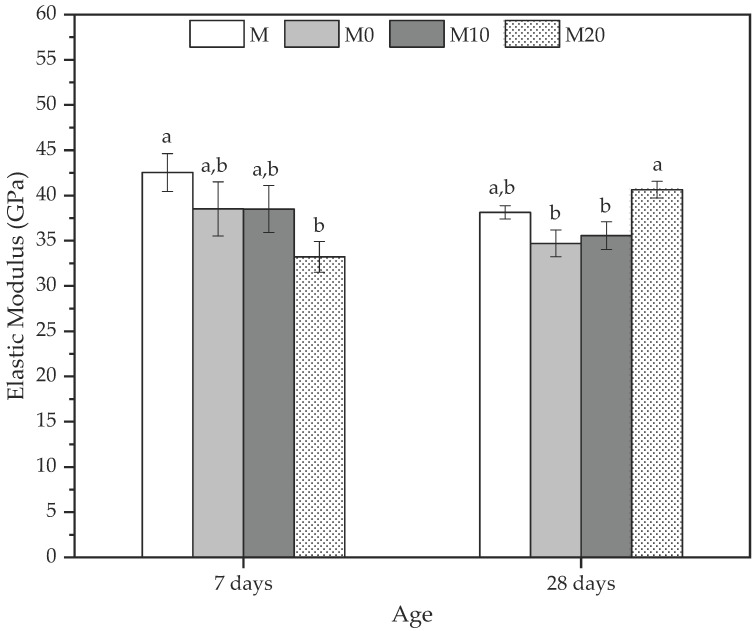
Elastic modulus at 7 and 28 days to mortars produced with 0, 10, and 20% Cloisite Na^+^ nanoclay hydrogels. Average with their respective standard deviation values, followed by equal letters do not differ statistically from each other following the Tukey test with a 95% confidence level.

A complementary method to analyze the mechanical properties of the mortars is through the elastic modulus (E-mod) determination at 7 and 28 d. Figure 7 shows the E-mod results for all mortars at the default ages.

In general, the behavior of all the mortars was similar to the dynamic elastic modulus discussed previously, and they agree with the compression strength results. At 7 d, reference mortar (M) presented the highest E-mod, indicating that the hydrogel influences the elastic behavior of these matrices. The reductions observed were 8.81%, 8.85%, and 27.04% for M0, M10, and M20, respectively. However, the statistical analysis indicated no statistically significant differences between the M, M0, and M10 mortars. At the same time, the values of M20 were statistically less than M, in agreement with Beushausen et al. [86]. They reported that using synthetic hydrogels remarkably reduces the modulus of elasticity of mortars. This mechanical behavior is similar to the dynamic elastic modulus, where the control samples presented higher values than M20, which had a reduction due to the water inside the hydrogel since the release from the polymer is slower and can cause a loss in stiffness [86].

The increase in the rigidity of the mortars with hydrogel, quantified by the E-mod, can be observed at 28 d, where the M20 presented an average value of 40.64 ± 0.92 GPa, corresponding to an increase of 16.55% in relation to reference mortar (M) due to the increase in the stress and the decrease in the strain presented by the mortar. These results also corroborate those for the compressive strength and dynamic elastic modulus, whereas the M20 was higher than other mortars because it improved their densification occasioned by efficient hydration provided by the hydrogels with the highest nanoclay concentrations.

### 3.8. Volume of Permeable Pore Space (Voids)

**Figure 8:**
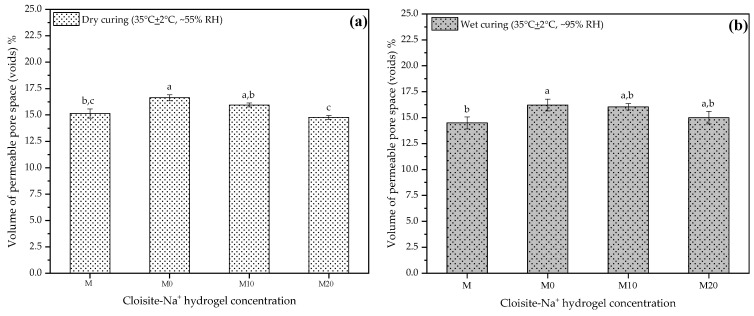
Volume of permeable pore space (voids) at 28 d to mortars produced with 0, 10, and 20% Cloisite Na^+^ nanoclay hydrogels, in two different curing conditions (**a**) dry curing and (**b**) wet curing. Average with their respective standard deviation values, followed by equal letters do not differ statistically from each other following the Tukey test with a 95% confidence level.

The volumes of permeable pore spaces (voids) at 28 d using the results from this study are presented in Figure 8a,b, respectively. These results are important and complementary to understanding some of the mechanical behavior observed previously because the desorption process by the hydrogel, over time, leads to the formation of voids in the microstructure, which plays a vital role in the mechanical properties [23]. Thus, each plot in Figure 8a,b represents the mean result for each mortar at two different curing conditions.

The results obtained were similar for the two curing conditions at 28 d, to which the samples were subjected before the test. Both M and M20 mortars presented a smaller pore volume, i.e., they did not present statistical differences between them. However, this behavior is more pronounced for samples placed in dry curing. For dry curing, mortars M0 and M10 showed an increase of about 9.91% and 5.28%, respectively, relative to the control. Under wet curing conditions, for these same mortars, the gains were 11.87% and 10.67%. This indicates that the release of water by the hydrogel is higher in hydrogels with smaller amounts of nanoclay in its composition, agreeing with what has been discussed by other researchers [32,35,56].

Thus, M20 has slower kinetics of release inside of cementitious matrix, which possibly maintains the internal moisture for a more extended period and consequently improves the hydration processes of the cement. Although the hydration products of the cement around the particles of the hydrogels can partially fill the voids occasioned by them, and thus tiny pores still can exist. The low resistance of the hydrogels introduces weak zones that decrease the mechanical performance of these mortars [21].

Moreover, even though they are still partially loaded with water inside the cement matrix, their high nanoclay concentration (20% wt/AAm + CMC wt) implies a more stable hydrogel structure, acting as a reinforcing agent and reducing the impacts of porosity on the mechanical mortar property.

All mechanical properties discussed in this study were impacted by hydrogel presence, and the results of the percentage of voids established this significant relation between mechanical properties and porosity. Nevertheless, this information helps design new mortar composites since voids are sites of weakness that control mechanical properties of these materials [87].

### 3.9. SEM-EDX Analysis

**Figure 9:**
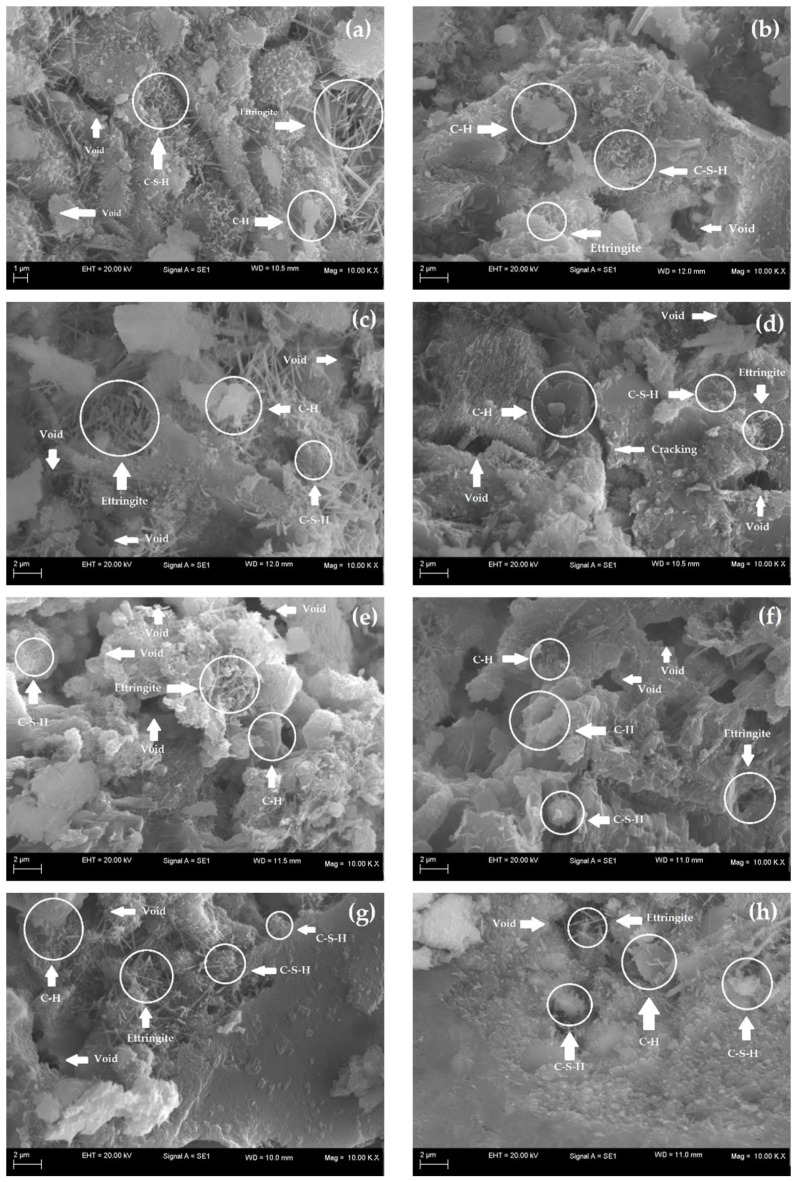
SEM images of the cement mortars (**a**) M (control) at 7 d, (**b**) M (control) at 28 d, (**c**) M0 at 7 d, (**d**) M0 at 28 d, (**e**) M10 at 7 d, (**f**) M10 at 28 d, (**g**) M20 at 7 d and (**h**) M20 at 28 d.

SEM analyses allow for characterization of the morphology of the microstructure of cementitious materials and thus evaluation of the effects of additions on the matrices of these materials. Therefore, to evaluate the impact of the use of nanocomposite hydrogels on the microstructures of the cementitious mortars, SEM images were selected for samples of mortars with and without hydrogels at 7 and 28 d, as shown in Figure 9a–g.

At 7 d, all samples present hydrated products with the extensive presence of needle-like ettringite crystals with a large crystal of portlandite (C-H). C-S-H can also be observed in all the samples, and its morphology is similar to a fibrous sponge, in agreement with Pourjavadi et al. [88]. However, it was not possible to identify the presence of hydrogels in M0, M10, and M20. This is possibly related to a low concentration of 0.5% wt of the presoaked hydrogel concerning the dry cement mass used in the mortar dosage. Thus, as described by Santos et al. [41], despite the lack of identification of the organic phase in the images, the mortars did not present incompatibility or phase separation, which is satisfactory as it indicates possible homogenization of the polymer in the cement matrix of the material.

At 28 d, the micrographs in Figure 9b,d,f,h illustrate denser matrices with smaller ettringite formations. However, the presence of nanocomposite hydrogels provides the establishment of a densified matrix due to the more efficient hydration of cement particles, especially for M20, as analyzed by Pourjavadi et al. [88]. In contrast, M0 and M10 showed voids and the appearance of some microfissures that directly contribute to their mechanical behavior [89,90]. In general, the formation of ettringite, C-H, and C-S-H occurs in all cementitious matrices, both the control (without hydrogel) and the ones containing hydrogels, indicating that the hardening reactions of the cement were effective over time, in agreement with Cilli et al. [91].

At 28 d, the cement matrix of M20 (Figure 9h) was more densified and similar to reference mortar M (Figure 9b), which is also seen in their similar mechanical behavior, indicating the efficiency of the hydrogels in the internal hydration process. The denser microstructures with few pores result from hydration and, consequently, the formation of more effective hydrated products [90]. Figure 9d,f also indicate that M0 and M10 at 28 d presented microstructures with voids and some microcracking, which reduced their compressive strength.

Table 5 presents the results obtained by applying the EDX technique but did not show significant changes for all the mortars studied. This technique was used to identify the presence of hydrogels in the cement matrix; however, the minimal or zero variation can be related to the low concentration of hydrogels used in the production of mortars. At 28 d, it was possible to verify that M0, M10, and M20 presented a small increase in the carbon element concerning the reference mortar (M); however, this variation was discrete, which does not allow us to conclude whether it is related to the presence of hydrogels or the occurrence of the carbonation process, as also observed by Santos et al. [41].

Thus, the other elements’ presence comes from the cement composition and the result of the formation of the hydration products, indicating that such reactions were effective over time.

## 4. Conclusions

This study investigated the effect of hydrogels on cementitious mortars’ fresh and hardened properties. The main findings are listed as follows:

- PAAm, CMC, and Cloisite-Na^+^ nanoclay nanocomposite hydrogel were successfully synthesized via free-radical polymerization. The Cloisite-Na^+^ concentration interferes directly in the hydrophilic property because the higher its concentration, the lower hydrogel swelling degree. It occurs because the free hydrophilic groups of the nanocomposite reduce with the Na^+^ increases, decreasing the difference in the osmotic pressure between the matrix and the swelling medium and, consequently, a retraction of the hydrogels with Cloisite-Na^+^;
- All nanocomposites, in distilled water medium, presented values of *n* above of 0.45 and bellow of the control matrix. The water absorption mechanism of all hydrogels has anomalous behavior, that is, when the diffusion times and relaxation rates of the chains are comparable. However, the increase in the Cloisite-Na^+^ concentration in the nanocomposite matrices modifies the water absorption, tending to Fickian transport, where the diffusion rate is much slower than the relaxation time of the polymer chain. This relaxation time is the time it takes for the chain to settle, that is, to come into balance with the presence of the solute or solvent;
- The swelling degree indicated the amount of water absorbed by the polymer. It allowed us to calculate the amount of water removed from the dosage to keep the water-cement ratio constant (w/c = 0.40). It was essential to avoid significant modifications in the mechanical properties;
- XRD patterns of hydrogel pure (PAAm and CMC) and hybrid nanocomposite hydrogels (PAAm/CMC and Cloisite Na^+^) confirmed that these matrices are predominantly amorphous, as expected due to their chains having high crosslinking density. However, it is possible to observe from hybrid hydrogels XRD that the characteristic nanoclay peak (2θ = 7.36°) shifted to the slight angle of the 2θ = 6.28°, causing an increase in basal spacing (d_001_ = 1.44 nm). This increase indicated the intercalation of nanoclay Cloisite Na^+^ in the polymer matrix.
- The decreases in the slump flow were 0.9%, 1.1%, and 4.9% for M0, M10, and M20 mortars, respectively, compared with the reference mortar (M). The reduction in free water of the fresh mixture will inevitably affect the workability of material cementitious. The effective w/c ratio is lower than that proposed by the initial dosage because part of the free water is stored in the absorbent polymer matrix. Despite reducing the workability of the mortar, the hydrogels filled with water will act as curing agents, improving the hydration of the cement particles within the cement matrix.
- In general, the results presented a slight variation among them, with a general average value of 99% in water retention in all mortars. This behavior can be related to the water parcel of dosage of hydrogel-mortars presoaked to reduce the availability of free water in the mix because this amount of water is stored, a priori, within the polymer particles.
- Cloisite Na^+^ acting as a reinforcing agent and modifying its absorption and water release kinetics also contributed to improvements in the mechanical properties at older ages. The increased nanoclay in the hydrogel matrix permits a more controlled water release over time and possibly resulted in better internal hydration. Additionally, the M20 system presented improved mechanical behavior with more extended aging.
- M and M20 presented lower percentages of voids in their structures; thus, their mechanical properties were similar and better than M0 and M10. This leads to the conclusion that the low resistance of the hydrogels introduces weak zones that decrease the mechanical performance of these mortars.
- The SEM images of all mortars present hydrated products with the extensive presence of needle-like ettringite crystals together with a large crystal of portlandite. Calcium silicate hydrate can also be observed in all samples, whose morphology is similar to a fibrous sponge. At 28 d, the micrographs represented denser matrices with smaller ettringite formations. However, the presence of nanocomposite hydrogel provides the establishment of a densified matrix due to more efficient hydration of cement particles, especially for M20.
- EDX did not show significant changes for all the mortars studied. However, minimal or zero variation can be related to the low concentration of hydrogel used in the production of mortars. At 28 d, it was possible to verify that M0, M10, and M20 presented a small increase in the carbon element concerning reference mortar; however, this variation was discrete, which does not allow us to conclude if it is related to the presence of hydrogel or the occurrence of the carbonation process.

Therefore, these hybrid nanocomposites are expected to bring new technology and improvements in the properties of cementitious material so that they can be applied in the future as efficient additives in the civil construction industry.

## Figures and Tables

**Table 1:** Chemical composition of cement Portland CPII-Z-32 [45].

Element	CaO	SiO_2_	SO_3_	Fe_2_O_3_	K_2_O	TiO_2_	SrO	MnO	Others
Composition (%)	75.29	16.19	3.70	3.11	1.11	0.25	0.24	0.04	0.07

**Table 2:** Physical properties of cement Portland CPII-Z-32 [46].

Loss on Ignition	Setting Time		Surface Area	Compressive Strength (MPa)			
(%)	Initial (min)	Final (min)	(m^2^/kg)	1 d	3 d	7 d	28 d
7.10	141	214	463.40	14.60	22.10	26.40	33.10

**Table 3:** Design of mix proportion of mortars samples.

Mix ID	CPII-Z-32 Cement	Siliceous Sand	Water (L/m^3^)			w/c Ratio		Hydrogel (%)	
	(kg/m^3^)	(kg/m^3^)	Dosage	Hydrogel ∗	Total	a/c_total_	a/c_effective_	Pre-Soaked **	Dry **
M	649.81	1403.59	259.93	-	259.93	0.40	-	-	-
M0	649.81	1403.59	256.77	3.16	259.93	0.40	0.39	0.50	0.015
M10	649.81	1403.59	256.80	3.13	259.93	0.40	0.39	0.50	0.018
M20	649.81	1403.59	256.81	3.12	259.93	0.40	0.39	0.50	0.021

* Amount of water corresponding to absorbed and stored water by the hydrogel. ** Percentage established in relation to the mass of cement used.

**Table 4:** Values of k and n obtained for different concentrations of Cloisite-Na^+^ in the hydrogels swelled in distilled water.

Cloisite-Na^+^ Concentration (% *w*/*w*)	Distilled Water			
	SD_eq_ (g·g^−1^)	*n*	*k* (h^−1^)	R^2^
0	34.707 ^a^	0.598 ^a^	0.154 ^a^	0.990
10	27.229 ^b^	0.546 ^a^	0.197 ^a^	0.983
20	24.389 ^c^	0.548 ^a^	0.198 ^a^	0.969

Average with their respective standard deviation values, followed by equal letters do not differ statistically from each other following the Tukey test with a 95% confidence level.

**Table 5:** EDX analysis for mortars produced with and without nanocomposite hydrogel.

Chemical Element	Reference M (% wt)		M0 (%wt)		M10 (%wt)		M20 (%wt)	
	7 d	28 d	7 d	28 d	7 d	28 d	7 d	28 d
C	4.26	3.41	3.51	4.17	4.10	4.43	4.32	3.98
O	53.30	54.84	54.92	55.30	58.93	49.97	53.60	50.36
Na	0.23	*	0.31	*	0.31	*	0.35	*
Mg	1.68	1.85	2.44	1.82	1.84	1.36	2.12	1.54
Al	1.88	1.77	2.41	2.54	2.50	0.77	2.04	1.75
Si	7.19	8.99	6.59	7.76	7.89	37.04	7.81	8.07
S	0.96	0.86	1.09	0.86	1.14	0.43	1.03	0.86
K	0.51	0.58	0.94	1.07	0.60	0.55	0.86	0.89
Ca	28.84	26.44	26.39	25.22	21.54	14.63	26.46	31.17
Fe	1.16	1.28	1.39	1.26	1.15	0.45	1.40	1.39

* Undetected element.

## Data Availability

Not applicable.
